# Single-pulse characterization of the focal spot size of X-ray free-electron lasers using coherent diffraction imaging

**DOI:** 10.1107/S1600577523000887

**Published:** 2023-03-22

**Authors:** Zichen Gao, Jiadong Fan, Yajun Tong, Jianhua Zhang, Bo He, Yonggan Nie, Hui Luan, Donghao Lu, Difei Zhang, Xinye Yuan, Yueran Wang, Zhi Liu, Huaidong Jiang

**Affiliations:** a School of Physical Science and Technology, ShanghaiTech University, 393 Middle Huaxia Road, Shanghai 201210, People’s Republic of China; b Center of Transformative Science, ShanghaiTech University, 393 Middle Huaxia Road, Shanghai 201210, People’s Republic of China; RIKEN SPring-8 Center, Japan

**Keywords:** X-ray free-electron laser, coherent diffraction imaging, spot size characterization, KB focusing

## Abstract

The characterization of X-ray focal spots is of great significance for the diagnosis and performance optimization of focusing systems. X-ray free-electron lasers (XFELs) are the latest generation of X-ray sources with ultrahigh brilliance, ultrashort pulse duration and nearly full transverse coherence. Because each XFEL pulse is unique and has an ultrahigh peak intensity, it is difficult to characterize its focal spot size individually with full power. Herein, a method for characterizing the spot size at the focus position is proposed based on coherent diffraction imaging. A numerical simulation was conducted to verify the feasibility of the proposed method. The focal spot size of the Coherent Scattering and Imaging endstation at the Shanghai Soft X-ray Free Electron Laser Facility was characterized using the method. The full width at half-maxima of the focal spot intensity and spot size in the horizontal and vertical directions were calculated to be 2.10 ± 0.24 µm and 2.00 ± 0.20 µm, respectively. An ablation imprint on the silicon frame was used to validate the results of the proposed method.

## Abstract 2

A new spot characterization method is proposed, based on coherent diffraction imaging that can accurately determine the focal spot size of a single X-ray free-electron laser pulse. This method was successfully applied to characterize the focal spot size at the Coherent Scattering and Imaging endstation of the Shanghai Soft X-ray Free Electron Laser Facility.

## 1. Introduction

The development of X-ray free-electron lasers (XFELs) offers unprecedented opportunities for X-ray science, including high temporal and spatial resolution imaging (Ayyer *et al.*, 2021 ▸; Jung *et al.*, 2021 ▸) and nonlinear interactions of X-rays with matter (Rohringer & Santra, 2007 ▸). XFELs have the advantages of high transverse coherence, high brightness and short pulse duration (Ackermann *et al.*, 2007 ▸; Ishikawa *et al.*, 2012 ▸; Kang *et al.*, 2017 ▸). There are more than 10^10^–10^12^ photons in a single pulse. With XFELs, frontier research at the atomic scale and femtosecond timescale for materials science and biology could be achieved by single-pulse experiments (Miao *et al.*, 1999 ▸; Neutze *et al.*, 2000 ▸; Chapman *et al.*, 2011 ▸). Coherent diffraction imaging (CDI) is a photon-starvation method (Gaffney & Chapman, 2007 ▸). It is a classic lens-less imaging method, proposed by Sayre in 1980 and realized by Miao in 1999 (Sayre *et al.*, 1980 ▸; Miao *et al.*, 1999 ▸). The image quality of CDI is affected by the luminous flux, noise and radiation damage (Howells *et al.*, 2009 ▸; Chapman & Nugent, 2010 ▸; Miao *et al.*, 2015 ▸). The femtosecond pulse of XFELs enables the collection of diffraction signals before the radiation damage process, which is called ‘diffraction-before-destruction’ (Chapman *et al.*, 2014 ▸). The excellent properties of the XFEL combined with CDI make high temporal and spatial resolution damage-free imaging possible. Currently, plane-wave CDI is widely used in imaging experiments at XFEL facilities (Takayama & Yonekura, 2016 ▸; Kobayashi *et al.*, 2021 ▸). Although the photon flux of each XFEL pulse is very high, a high coherent photon flux density is also required to achieve high resolution. Usually, Kirkpatrick–Baez (KB) mirrors are used to focus the XFEL beam to increase the photon density at the sample position. The focal spot size usually ranges from a few micrometres to tens of nanometres.

For plane-wave CDI, a large spot size is generally required to illuminate the entire sample. Therefore, the spot size limits the sample size (Chapman & Nugent, 2010 ▸). In addition, the size of the XFEL spot at the sample position is an important indicator of focusing accuracy. There are well established applications for synchrotron facilities, such as photoelectric imaging (Sun *et al.*, 2006 ▸), wire scanners (Shao *et al.*, 2016 ▸) and synchrotron radiation interferometry (Hayano *et al.*, 1999 ▸). However, owing to the instability of the XFEL, each XFEL pulse is unique, particularly for self-amplified spontaneous emission (Saldin *et al.*, 1995 ▸; McNeil & Thompson, 2010 ▸). Therefore, measurement of the XFEL focal spot is required shot by shot. As the peak intensity of each XFEL pulse is very high, detectors can be damaged, and most spot-size measurement methods used in synchrotron facilities are no longer suitable. There are already a few methods to characterize the XFEL spot size, such as knife-edge scanning, damage detection on the substrate and Hartmann wavefront sensors (Koyama *et al.*, 2013 ▸; Pikuz *et al.*, 2015 ▸; Keitel *et al.*, 2016 ▸; Hua *et al.*, 2019 ▸). For example, the knife-edge scan method is widely used to measure the spot size at the focus position. To avoid radiation damage to the knife edge, the peak intensity is usually attenuated down to the radiation damage threshold, making it impossible to measure the real focus at full peak intensity. A result calculated from multiple pulses is obtained, that includes the error caused by spot instability. Hartmann wavefront sensors provide an advanced method for XFEL wavefront detection and beam characterization. However, the Hartmann wavefront sensor must be calibrated with an ideal wavefront to obtain the real spot size, and its results are affected more by systematic errors.

Here, we propose a simple but accurate method called the reconstruction method to characterize single-shot XFEL spot sizes at the focus position using CDI. The single-shot focal spot size can be quickly and accurately calculated from the reconstructed image by recording single-shot diffraction patterns and taking the phase retrieval of the diffraction pattern. Gold nanospheres and nanocubes were used to demonstrate the validity of the proposed method. Single-shot diffraction patterns from the tested samples were collected and reconstructed into images. We then used the Gaussian distribution to estimate the light spot according to the position and intensity distribution of the reconstructed samples, and acquired the XFEL spot size by two-dimensional intensity and beam fitting. A simulation was performed to verify the proposed method. The experiment was performed at the Coherent Scattering and Imaging (CSI) endstation of the Shanghai Soft X-ray Free Electron Laser Facility (SXFEL).

There are already some methods for characterizing the spot through analysis of the speckle or fluorescence. Spatio-temporal coherence, energy and spot size of XFELs have been analyzed by the scattering of single or multiple particles (Inoue *et al.*, 2015 ▸; Yun *et al.*, 2019 ▸; Lee *et al.*, 2020 ▸; Nakamura *et al.*, 2020 ▸). These methods are meaningful in XFEL pulse diagnosis. Most of them provide a statistical result, while our method can characterize a single XFEL pulse during the imaging experiment and does not require a special experimental setup.

## 2. Simulation

### 2.1. Reconstruction method

When a coherent beam illuminates a sample, the diffraction amplitude in the far field is the Fourier transform of the light wavefront multiplied by the sample electron density distribution, and the diffraction pattern intensity is the amplitude multiplied by its conjugate, as shown in equation (1),

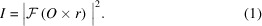

**Figure 1:**
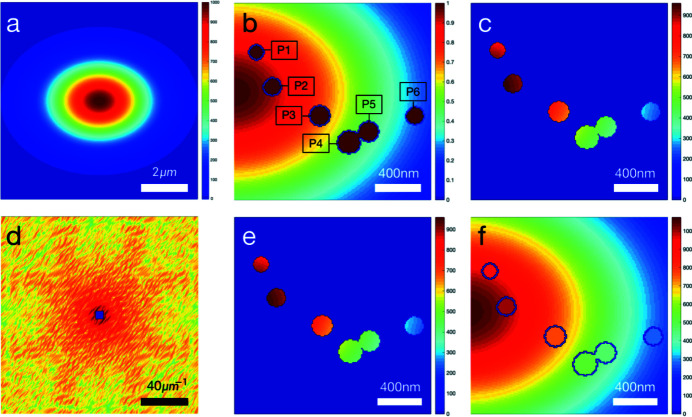
Characterization of spot size from simulated diffraction patterns of uniform disk-shaped particles under the assumption of a fully coherent Gaussian beam. (*a*) Gaussian beam amplitude distribution with a 2.272 µm FWHM on the *x* axis (FWHM_*x*_), a 1.704 µm FWHM on the *y* axis (FWHM_*y*_) and a peak amplitude of 1000 (arbitrary units). The beam pattern has 500 × 500 pixels. (*b*) Sample composed of six disk-shaped particles having the same thickness. The sample is marked with blue circles and the sample total density has been normalized. The background outside the blue circles is the normalized spot amplitude, which shows the relative positions of the spot and the sample. (*c*) The sample illuminated by Gaussian beam. The intensity of the illuminated sample is equal to the original intensity of the sample multiplied by the spot amplitude and the background is zero. (*d*) The diffraction pattern, which has 500 × 500 pixels, and to which 5% Poisson noise and 20 × 20 pixels missing data in the center have been added. (*e*) Reconstruction of the particles. The HIO and ER algorithms were used iteratively. The result is the average of the 100 groups with the smallest error selected from the 1000 groups used for reconstruction results. (*f*) Result of spot fitting. The background outside the blue circles shows the fitted spot amplitude and the blue circles mark the reconstructed particles.

Here, *I* is the intensity of the diffraction pattern, *O* is the light amplitude modulus distribution at the sample plane, *r* is the sample electron density distribution, and 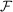 is the Fourier transform operator. The intensity distribution at the focal spot of the KB mirror can be described by a two-dimensional Gaussian function, as shown in Fig. 1 ▸(*a*) (Self, 1983 ▸). The long and short axes of the spot should be in the horizontal (*x*) or vertical (*y*) directions because the spot is focused by horizontal and vertical KB mirrors. We used six uniform disk-shaped particles with different diameters for the simulation [Fig. 1 ▸(*b*)]. The sample was illuminated with a Gaussian beam, which had a spot size ω [full width at half-maximum (FWHM) of light intensity] of 1.606 µm in the horizontal direction (ω_*x*_) and 1.205 µm in the vertical direction (ω_*y*_). Its amplitude had a FWHM in the horizontal and vertical directions of FWHM_A*x*_ = 2.272 µm and FWHM_A*y*_ = 1.704 µm, respectively [Fig. 1 ▸(*a*)]. The spot size is the FWHM of the light intensity, and the light intensity is proportional to the square of the amplitude. The spot size ω = 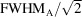 [Fig. 1 ▸(*c*)] shows the product of light amplitude modulus and sample electron density at each pixel. In addition, 5% Poisson noise and 20 × 20 pixels of missing data were added to the pattern [Fig. 1 ▸(*d*)]. 2800 iterations of the hybrid input–output (HIO) and 200 iterations of the error reduction (ER) algorithm were used for reconstruction, and the results are shown in Fig. 1 ▸(*e*).

The light amplitude at the center pixels of the particle can be approximated to have a uniform variation. The intensity at each pixel in the particle reconstruction was proportional to the light amplitude at that pixel multiplied by the number of electrons in the pixel. All particles were entirely composed of gold, and thus had similar electron densities. The number of electrons in each pixel was proportional to its volume or thickness. The beam intensity at each pixel can be described by the equation

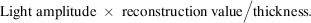

Since the intensity at the edge pixels in the reconstruction might be inaccurate, we selected 3 × 3 pixels near the center of each particle to calculate the light amplitude at the center. We normalized the thicknesses of the disk-shaped particles and calculated the light amplitude at the center and the position of each particle (Table 1 ▸).

The amplitude modulus of the Gaussian beam at the focal plane with the major and minor axes in the *x* (horizontal) or *y* (vertical) directions can be described by the following equation,

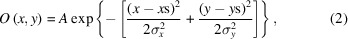

where *A* is the peak amplitude, (*x*s, *y*s) is the position of the spot center, and σ_*x*_ and σ_*y*_ are the standard deviations which reflect the spot size. These five quantities (*A*, *x*s, *y*s, σ_*x*_, σ_*y*_) were fitted. Therefore, at least five particles were required to determine the light distribution. From the reconstruction results, we can obtain the approximate area of the center of the spot and the FWHM_A_ range. These estimates were used as the initial value and range of fit. The relative light amplitude (RA) is the average light intensity of 3 × 3 pixels near the center. RA is proportional to actual light amplitude.

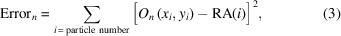




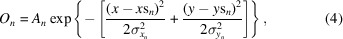




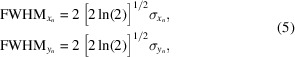




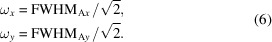

The subscript *n* means different experiment times. We find the approximate ranges of (A_*n*_, *x*s_*n*_, *y*s_*n*_, σ_*xn*_, σ_*yn*_) from the reconstruction and fit them using least squares. Then, the spot dimensions ω_*x*_ and ω_*y*_ can be obtained. The comparison of the fitted spot with the reconstruction of the particles is shown in Fig. 1 ▸(*f*). The agreement of the fitting result with the set value (shown in Table 2 ▸) indicates that the reconstruction method is accurate for determining the spot size.

### 2.2. Sample requirement

**Figure 2:**
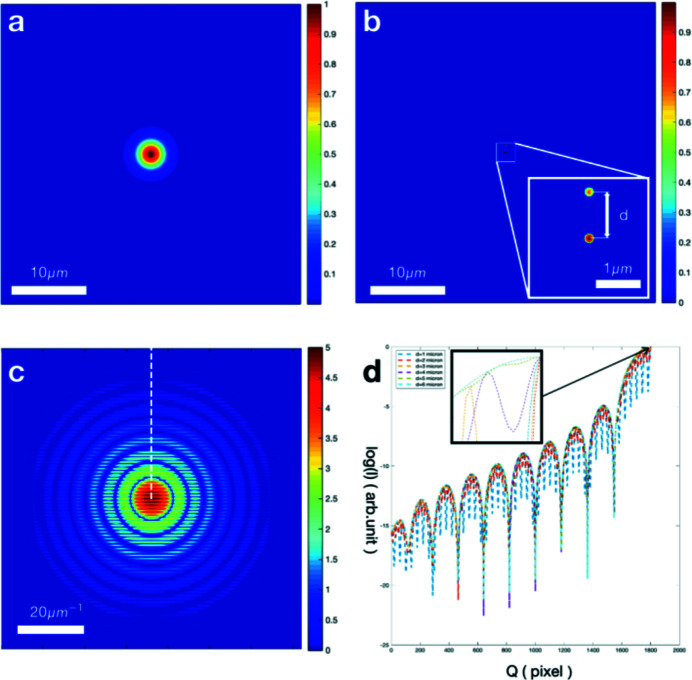
Determination of the largest size of the sample by observing the interference fringes. (*a*) Circular Gaussian beam amplitude. The spot has a size ω = 2.12 µm, 3 µm FWHM_A_ and a peak amplitude of 1 (arbitrary units). The pattern has 3605 × 3605 pixels. (*b*) Sample multiplied by beam amplitude. The sample is composed of two identical gold nanoparticles with 200 nm diameter; one is at the center of the spot and the other is at a distance *d*. (*c*) Logarithmic diffraction intensity pattern from the sample in (*b*) illuminated by the beam in (*a*). (*d*) Line scan of the white dotted line in (*c*). The normalized line scan results with different *d* show interference fringes.

The reconstruction method has requirements for samples. If the particles in the sample are too far apart, they cannot be reconstructed. Here, we simulate using parameters close to the actual experiment: circular Gaussian beam, FWHM of beam amplitude FWHM_A_ = 3 µm, spot size ω = 2.12 µm [Fig. 2 ▸(*a*)]. The sample consists of two identical gold nanoparticles with 200 nm diameter; one is at the center of the spot and the other is at a distance *d* [Fig. 2 ▸(*b*)]. As *d* increases, interference fringes in the intensity pattern become less visible [Figs. 2 ▸(*c*) and 2(*d*)]. Fig. 2 ▸(*d*) shows that the interference fringes are almost invisible when *d* is greater than 5 µm, and the sample cannot be reconstructed correctly. The spot size in the experiment is about 2 µm, so the total size of the sample cannot exceed 5 µm. In addition to the limitation on the sample size, there is also a limitation on the number of gold nanoparticles in the sample. More particles will increase the saturation area and the difficulty of finding support. In the future, samples with limited size and particle number will make experiments more reliable.

## 3. Experiments

### 3.1. Reconstruction method

**Figure 3:**
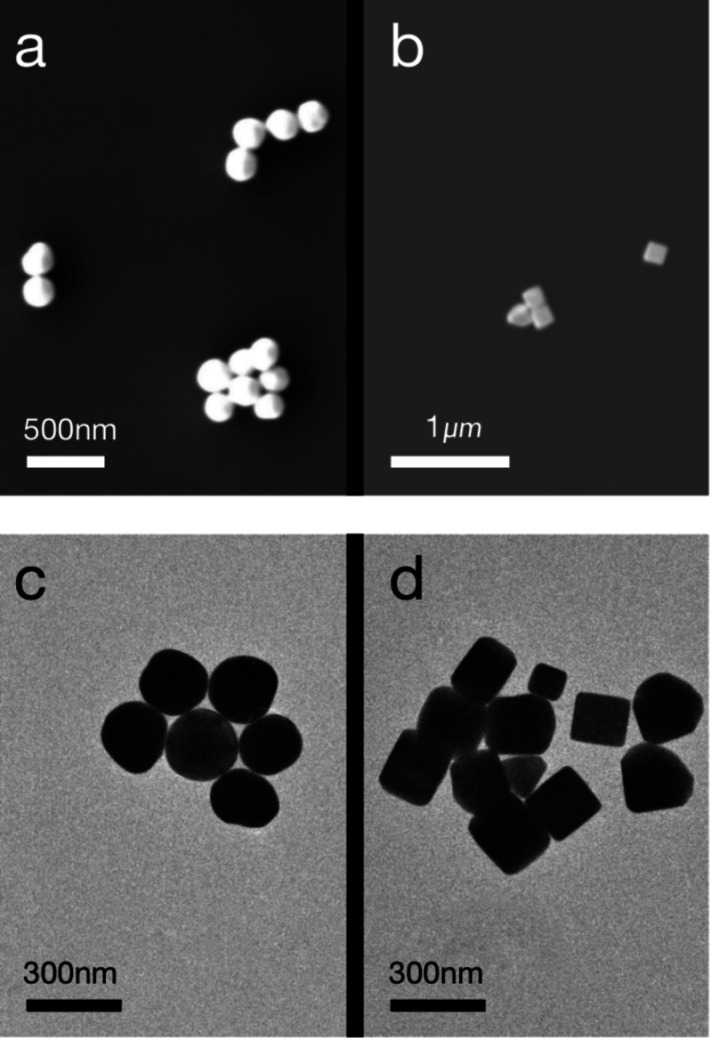
Gold nanoparticles for the experiments. (*a*) SEM image of gold nanospheres of diameter 200 nm. (*b*) SEM image of gold nanocubes with an average edge length of about 170 nm. (*c*) TEM image of gold nanospheres with similar density. (*d*) TEM image of gold nanocubes.

The CSI endstation is one of the five endstations at SXFEL. It can carry out forward X-ray scattering experiments such as CDI, Fourier transform holography and small-angle X-ray scattering (Fan *et al.*, 2022 ▸). Combined with a pump laser, it is possible to obtain dynamic structures of materials with femtosecond temporal resolution. Our experiment was performed at the CSI endstation of SXFEL. The SXFEL is a soft X-ray free-electron laser, with photon energies ranging from 100 eV to 620 eV. At the oxygen absorption edge, the X-ray photon energy was set to 520 eV. Two types of nanoparticles were imaged, including 200 nm gold nanospheres and 170 nm gold nanocubes. Scanning electron microscope (SEM) and transmission electron microscope (TEM) images of the 200 nm gold nanospheres are shown in Figs. 3 ▸(*a*) and 3(*c*), respectively. The SEM image shows a uniform size distribution. The TEM images show that the densities of the nanospheres varied because of incomplete crystallization. Figs. 3 ▸(*c*) and 3(*d*) show SEM and TEM images of the nanocubes, whose size and shape distributions were larger than those of the nanospheres. Similar to the nanospheres, the TEM image shows that the nanocubes also had different densities. The nanoparticles were deposited on Si_3_N_4_ membranes with a thickness of 50 nm. The Si_3_N_4_ membranes were mounted on two-dimensional scanning stages. By synchronizing the scanning stages and pulse picker with trigger signals, the single-shot diffraction pattern can be recorded by an X-ray detector (model PI-MTE3: 4096B-2), which has 15 µm × 15 µm pixel size and 4096 × 4096 pixels. To protect the detector from direct beams, beamstops of various diameters can be moved into the beam path. For our experiment, a 2 mm-diameter beamstop was used, and the detector was mounted 250 mm downstream of the sample.

**Figure 4:**
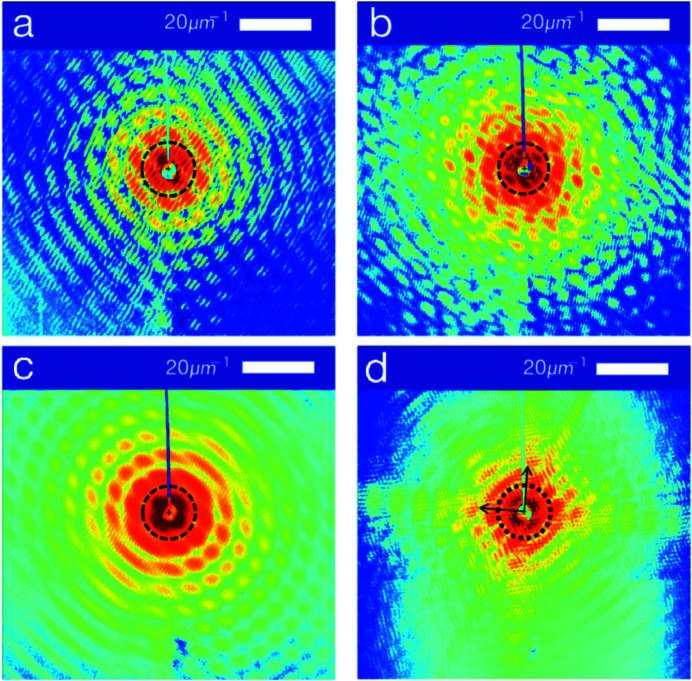
Panels (*a*)–(*d*) show four patterns from different particles used for reconstruction, where (*d*) represents the gold cube and the rest represent the gold spheres. The black dashed circles in (*a*)–(*d*) represent the size of the zeroth-order diffraction spots and the black arrows in (*d*) represent the diffraction direction of the main particle. The blank parts at the top of the patterns are missing data. A beamstop with 2 µm diameter was applied in the center of the detector. The signals blocked by the beamstop and its rod cause missing data. The pattern had 3605 × 3605 pixels and the detector pixel size was 15 µm. The background has been subtracted from each pattern. The background is a pattern without illuminated particles. A 5 × 5 binning with 721 × 721 pixels was applied to the patterns used for reconstruction.

Single-shot diffraction patterns were recorded using an X-ray detector by scanning the Si_3_N_4_ membranes deposited with nanoparticles using X-ray pulses. The diffraction patterns of the gold nanospheres are shown in Figs. 4 ▸(*a*)–4(*c*). The black dashed circles depict the characteristic diffraction rings. The clear interference to the side of the diffraction spots indicates that multiple nanospheres were imaged within a single pulse. For each XFEL pulse, there were approximately 10^12^ photons, corresponding to approximately 100 µJ of energy (Fan *et al.*, 2022 ▸). Owing to the ultrahigh flux intensity, the diffraction signals extend to the corners of the X-ray detector. The maximum spatial frequency was approximately 80 µm^−1^, corresponding to a half-period resolution of 6.25 nm. Fig. 4 ▸(*d*) shows diffraction patterns from the gold nanocubes. Compared with the diffraction patterns of the gold nanospheres, diffraction fringes were observed instead of diffraction rings, as indicated by the black arrows.

**Figure 5:**
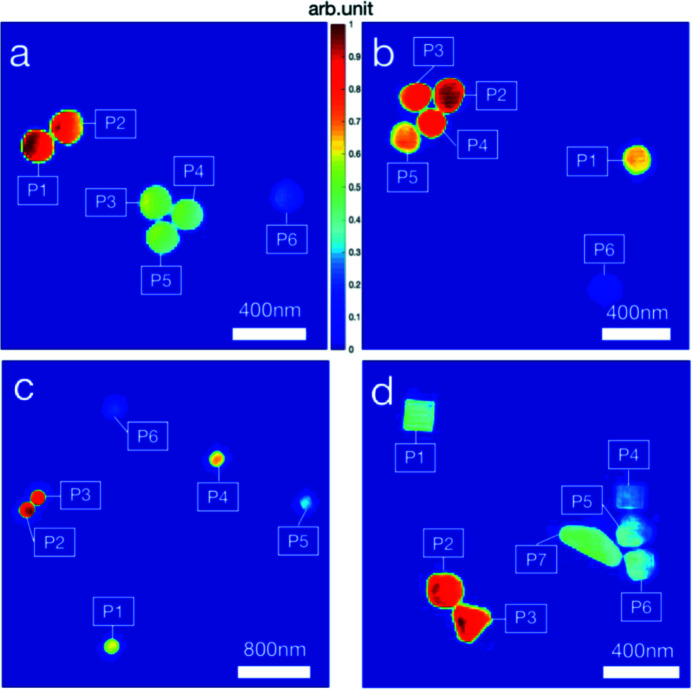
Normalized reconstructions of the patterns in Figs. 3 ▸(*a*)–3(*d*). The pixel size is 11.1 nm. The white labels mark the particles in each reconstructed image.

The images of the samples were reconstructed using a phase retrieval algorithm that included 2800 HIO iterations followed by 200 ER iterations. For each diffraction pattern, 1000 independent reconstructions were conducted. Among these, 100 independent reconstructions with the smallest reconstruction errors were averaged to obtain the final image. The reconstructed images are shown in Fig. 5 ▸. Because reconstruction is the product of gold nanoparticle densities and beam intensity distribution, the reconstructed intensities were unequal for each nanoparticle.

**Figure 6:**
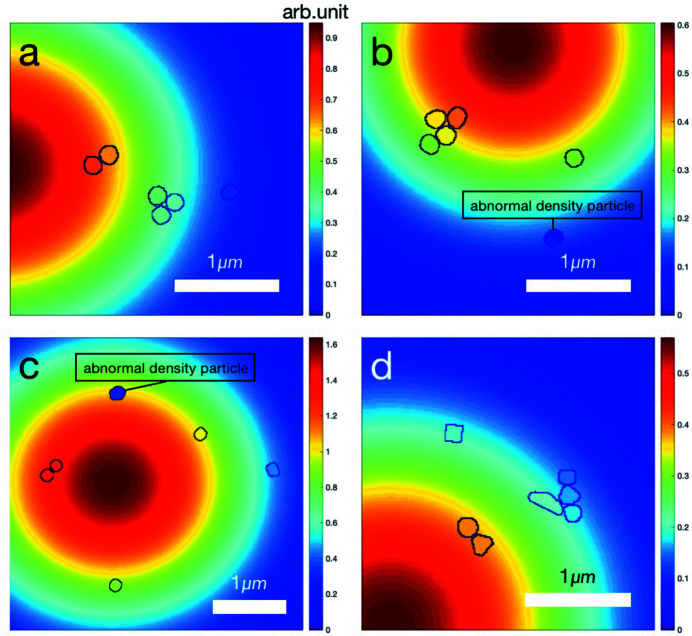
Results of spot fitting. The blue circles mark the reconstructed particles and the background outside the blue circles shows the fitted spot amplitude. The black labels mark the abnormal density particles, which were not used for spot fitting.

As shown earlier, the beam spot size can be calculated from the reconstructed images. The reconstructed images were expanded to 1000 × 1000 pixels by padding with zeros. The position and average center intensity of each nanoparticle were calculated, as shown in Table 3 ▸. Owing to incomplete crystallization, as shown in Fig. 3 ▸, the densities of a few nanoparticles were found to be abnormal. These low-density particles were not used in the fitting process. The particle sizes are listed in Table 3 ▸. By omitting nanoparticles with abnormal density, the spot-fitting method introduced in the simulation section was applied to the reconstructed images. The distribution of each nanoparticle in the beam profile is shown in Fig. 6 ▸. The fitted single-shot spot sizes are presented in Table 4 ▸.

The spot size variation was calculated to be ω_*x*_ = 2.10 ± 0.24 µm and ω_*y*_ = 2.00 ± 0.20 µm. The advantage of the reconstruction method was the online single-shot characterization of focal spot size. In addition, the damage method, described below, was used to verify the fitting results of the reconstruction method.

### 3.2. Damage method

**Figure 7:**
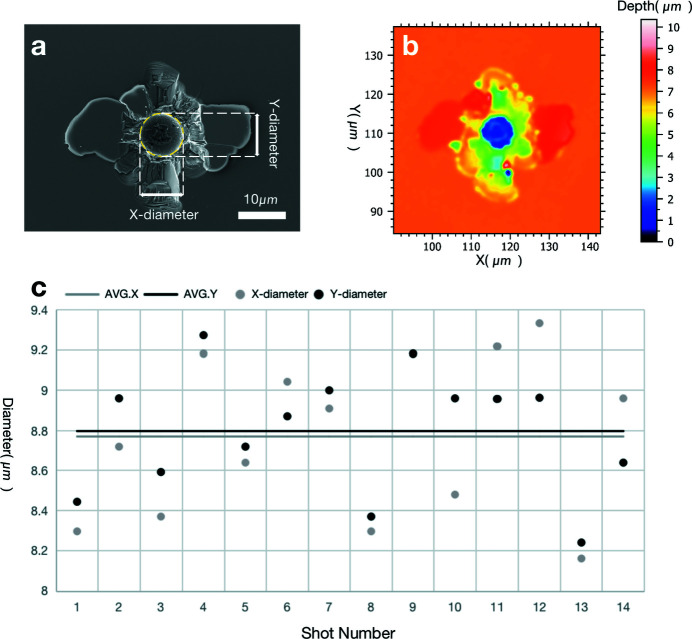
(*a*) SEM image of one typical damaged area on the silicon wafer. Its ablation area is marked with a yellow circle. The two white lines show the *x* and *y* diameters of the ablation area. (*b*) 3D-CLSM image of the damaged area shown in (*a*); the deepest part is about 7 µm from the surface. (*c*) The *X* and *Y* diameters of the 14 ablation areas are measured and displayed as points. The two straight lines are the average values of the *X* (gray line) and *Y* (black line) diameters.

The silicon wafer coated with a 50 nm-thick layer of Si_3_N_4_ was irradiated with a high-fluence focused XFEL pulse without attenuation. Fig. 7 ▸(*a*) shows a typical damaged area on the silicon wafer. The circular crater in the center is an ablation area, while the surface cracks may be caused by the ejection of evaporated silicon. We counted 14 ablation areas, and Fig. 7 ▸(*c*) shows their *x*- and *y*-axis diameters. The diameters of the ablation areas along the *x* and *y* axes are *D*_*x*_ = 8.77 ± 0.40 µm and *D*_*y*_ = 8.79 ± 0.31 µm. We then used a 3D confocal laser scanning microscope (3D-CLSM) to detect the damaged area, shown in Fig. 7 ▸(*b*). The depth of the ablation area was approximately 7 µm. The edge of the ablation area was slightly below the surface, because of which the calculated diameter was smaller than the true value. The calculated spot size was verified by evaluating the ablation area. The size of the ablation area is related to the energy density of the light and ablation threshold of the material. The threshold fluence of silicon is given by

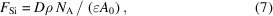

where ɛ, *A*_0_, ρ, *N*_A_ and *D* are the absorption coefficient, average atomic weight, average density, Avogadro’s constant and melting dose, respectively (Koyama *et al.*, 2013 ▸). The threshold fluence of silicon for a wavelength of 2.4 nm is *F*_Si_(λ = 2.4 nm) = (6.61 ± 0.36) × 10^−3^ µJ µm^−2^. A maximum pulse energy greater than 200 µJ was achieved at the source point, and the pulse energy at our estimation is about 100 µJ (Fan *et al.*, 2022 ▸). The beam condition of SXFEL is not stable enough. Thus we set the energy threshold through the gas intensity monitor detector reading. The pulse arriving at the sample point is the pulse after a high-energy pulse, which usually has high energy (∼100 µJ) (Tong *et al.*, 2022 ▸). However, the 50 nm-thick Si_3_N_4_ layer will attenuate it by 20%. The energy absorbed by Si was approximately 80 µJ. The energy density distribution of the spot, Φ(*x*, *y*), is approximately given by

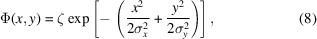




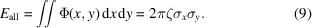

Here, *E*_all_ = 80 µJ, σ_*x*_ = ω_*x*_/2[2ln(2)]^1/2^, σ_*y*_ = ω_*y*_/2[2ln(2)]^1/2^. So the coefficient ζ is given by

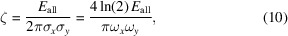




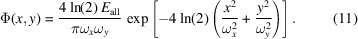

When the beam energy density reaches the threshold fluence, Φ(*x*,*y*) = *F*_Si_(λ = 2.4 nm) and

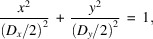

and ω along the *x* and *y* axes are ω_*x*_ = 2.71 ± 0.14 µm, ω_*y*_ = 2.72 ± 0.11 µm, respectively. These values are in good agreement with the measured values shown in Table 4 ▸. The spot size calculated from the damage is highly dependent on the pulse energy. Due to the pulse energy fluctuation of SASE mode, the pulse energy of each shot cannot be absolutely measured during the experiments. This method uses 100 µJ for estimation, which is not accurate for the calculation of spot size. However, the ratio of the spot size in the *x* and *y* directions can be obtained from this method, which shows that the spot is close to circular.

## 4. Conclusion

The reconstruction method based on coherent diffraction imaging requires only a single pulse, and it can accurately characterize the spot, which provides an *in situ* real-time analysis approach for future experiments. This is also helpful for diagnosing and optimizing light sources online. Using the experimental data from the CSI endstation, the reconstruction method shows that the spot intensity at the focal point had a 2.1 µm horizontal FWHM and 2.0 µm vertical FWHM. The result obtained by the reconstruction method is consistent with that of the damage method and knife-edge scanning method (Tong *et al.*, 2022 ▸). Therefore, the reconstruction method is feasible for characterization of the XFEL spot. This result is close to the minimum size of the SXFEL focus in theory, indicating that the optimization of the optical path is excellent.

## Figures and Tables

**Table 1:** Center position and relative light amplitude (RA) of each particle The particle number is labeled in Fig. 1 ▸(*b*). RA is the average light amplitude of 3 × 3 pixels near the center, and is proportional to the actual light amplitude.

Particle	(*x*, *y*, RA)
P1	(271.00, 221.00, 876.30)
P2	(283.09, 247.11, 932.67)
P3	(319.00, 269.00, 708.28)
P4	(341.09, 289.11, 493.98)
P5	(356.00, 281.00, 433.77)
P6	(391.09, 269.11, 263.04)

**Table 2:** Actual value and fitting results for *x*s, *y*s, FWHM_A*y*_, FWHM_A*y*_, ω_*x*_, ω_*y*_ and *A* (*x*s, *y*s) is the position of the spot center. The fitting results are the averages of the results with the smallest errors.

	Actual value	Fitting result
*x*s	251	248.8
*y*s	251	251.6
FWHM_A*x*_ (µm)	2.272	2.298
FWHM_A*y*_ (µm)	1.704	1.714
ω_*x*_ (µm)	1.606	1.625
ω_*y*_ (µm)	1.205	1.212
*A* (arbitrary units)	1000	1009

**Table 3:** Center position and relative light amplitude of each particle in each reconstruction [Figs. 5 ▸(*a*)–5(*d*)] Particles P6 in (*b*) and P6 in (*c*) (highlighted in bold) are abnormal density particles. These two particles were omitted from the inputs for fitting.

Particle data	*a*(12682) (*x*, *y*, RA)	*b*(23445) (*x*, *y*, RA)	*c*(24135) (*x*, *y*, RA)	*d*(15654) (*x*, *y*, RA)
P1	(484.42, 510.41, 0.72)	(508.08, 496.31, 0.32)	(498.40, 503.76, 0.89)	(482.34, 405.45, 0.20)
P2	(498.03, 501.63, 0.65)	(407.84, 461.81, 0.43)	(414.19, 368.24, 1.37)	(494.26, 485.83, 0.38)
P3	(539.54, 536.58, 0.42)	(389.93, 462.92, 0.38)	(425.33, 356.28, 1.34)	(505.96, 500.33, 0.39)
P4	(554.35, 542.20, 0.36)	(398.64, 477.05, 0.37)	(602.85, 317.57, 1.00)	(579.10, 442.90, 0.15)
P5	(542.77, 553.10, 0.39)	(384.72, 484.59, 0.31)	(691.00, 361.44, 0.45)	(580.66, 458.63, 0.16)
P6	(601.00, 533.62, 0.18)	**(490.67, 564.74, 0.05)**	**(500.77, 267.25, 0.26)**	(583.74, 473.67, 0.18)
P7				(561.50, 464.22, 0.21)

**Table 4:** Reconstruction fitting results The data are the averages of the results with the smallest errors, plus or minus one standard deviation. (*x*s, *y*s) is the position of the spot center. The average value of the five results for FWHM_A_ and ω along the *x* and *y* axes is also shown.

	*x*s	*y*s	FWHM_A*y*_ (µm)	FWHM_A*y*_ (µm)	ω_*x*_ (µm)	ω_*y*_ (µm)	*A* (arbitrary units)
*a*(12682)	398.7	511.4	2.95	2.61	2.09	1.85	0.96
*b*(23445)	455.5	397.9	2.51	2.56	1.77	1.81	0.61
*c*(24135)	494.4	376.5	3.25	3.01	2.30	2.13	1.64
*d*(15654)	427.5	569.2	3.18	3.11	2.25	2.20	0.57
Average			2.97 ± 0.33	2.82 ± 0.28	2.10 ± 0.24	2.00 ± 0.20	
